# The Humoral Theory of Transplantation

**DOI:** 10.1155/2017/5935123

**Published:** 2017-08-08

**Authors:** Mepur H. Ravindranath, Junchao Cai, Soldano Ferrone, Frans H. J. Claas, Senthamil R. Selvan

**Affiliations:** ^1^Terasaki Research Institute, Los Angeles, CA 90064, USA; ^2^Massachusetts General Hospital, Harvard Medical School, Boston, MA 02114, USA; ^3^Eurotransplant Reference Laboratory, Leiden University Medical Centre, 2333 ZA Leiden, Netherlands; ^4^Omni Array Biotechnology, Rockville, MD 20855, USA

In 2003, after 45 years of research on the humoral theory of donor organ rejection, the late Professor Paul Ichiro Terasaki (09/10/1929–01/25/2016) proposed the Humoral Theory of Transplantation in the American Journal of Transplantation (3:665–673, 2003) and continued doing his research until his last breath. We respectfully dedicate this special issue in memory of Professor Paul I. Terasaki, the founding father of Humoral Theory of Transplant Immunology.

His theory not only impacts his contemporaries to develop better therapeutic strategies but also directs generations that follow him.

The graft of an organ from a living or deceased donor to a recipient causes many immunological reactions, which in many cases results in the failure of the allograft within the recipient's body. Whether grafts are destroyed by direct cytotoxicity, mediated by cellular immune components such as T cells, NK cells, or delayed-type hypersensitivity reactions, or by antibodies is the critical question. The ability of antibodies to destroy an organ within a few hours is well known; an example of this is the transfusion-related acute lung injury (TRALI). These antibodies can cause immediate rejection (even before closing the incision or soon afterwards) in hyper acute rejection, acute rejection (within a year), and chronic rejection (a year after posttransplant). The involved antibodies recognize either alloantigens such as cell surface MHC antigens (primarily donor-recipient mismatched HLA class I and class II antigens, known as donor specific antibody (DSA)) or normal or altered autoantigens such as endothelial antigens, angiotensin II type 1 receptor (AT1R), antiendothelin-I type A receptor (ET_A_R), bioreactive C-terminal fragment of perlecan, vimentin, collagen V, K-a1 tubulin, and myosin to name a few. The primary objective of the proposed humoral theory is to provide a logical and rational course of clinical strategy for all allograft recipients. Antibody-mediated allograft deterioration is also considered an antibody-accelerated allograft senescence. The need to update clinical and experimental findings on antibody-mediated graft rejection and to explore the precise mechanisms underlying humoral rejection in designing therapeutic strategies to prevent allograft deterioration have prompted us to organize this issue.

We wish to profusely and individually thank every one of the contributors for positively responding to our request to memorialize the famous humoral theory of Dr. Paul Terasaki in the Journal of Immunology Research. The manuscripts received are categorized as research articles (*n* = 9), clinical studies (*n* = 2), and reviews (*n* = 10).

The special issue includes the following research articles: A. I. Sánchez-Fructuoso et al. (*Hospital Clinico San Carlos (IdiSS) Madrid, Spain*) documented that a shift in a single-nucleotide polymorphism (SNP: with a shift at position −308 from G to A) augmented the production of TNF*α* and is correlated with a significantly increased risk of acute rejection. This SNP is found to be a predictive biomarker of the efficacy of thymoglobulin, commonly used as an immunosuppressive regimen for lowering antibody production. Y. Jiang et al. (*The First Affiliated Hospital, Zhejiang University, China*) examined the infiltration of CD20+ B cells and C4d in biopsies of patients (*n* = 216) with biopsy-proven acute cellular rejection (Banff I or II), in addition to serum creatinine and glomerular filtration rates to assess graft loss. In contrast to previous reports, they documented that the CD20+ patients (n-133) had significantly less graft loss and a better (but not a significant) survival rate and less steroid resistance than the CD20-negative group. It is concluded that the presence of CD20+ B cells in allografts is protective. J. C. Cicciarelli et al. (*USC Keck School of Medicine, Viracor-IBT Laboratories, MNTI Foundation, Los Angeles, CA*) analysed C1q and IgG subclasses in 73 renal allograft recipients for graft dysfunction with DSA. In analysing the graft biopsies for C4d, a remarkable difference was observed in cumulative DSA MFIs between the C4d+ group (12,500) and the CD4− group (<500). Among the C4d+ biopsy groups, 100% had DSA IgG, 85% had complement-fixing IgG, and 70% had C1q but did not observe any significant correlation between graft loss and C1q positivity. X. Zhao et al. (*Peking University People's Hospital, Beijing, China*) examined the association between anti-HLA DSA and prolonged isolated thrombocytopenia (PT) in a large cohort of unmanipulated haploidentical blood and bone marrow transplant (HBMT) patients (*n* = 394). The incidence of PT is significantly higher in patients with high MFIs (>1000) than those with low MFIs (<1000). Multivariate analysis revealed a significant correlation between high MFIs and hazard ratios of PT and, most importantly, a significant transplant-related mortality, thus emphasizing the need to include DSAs in the algorithm of unmanipulated HBMT. M. Toyoda et al. (*Cedars-Sinai Medical Center, Los Angeles, CA, USA*) report that IVIg plus rituximab, in combination with alemtuzumab and triple immunosuppression maintenance therapy, does not increase the risk of viral (CMV, EBV, and BKV) infections based on posttransplant viral infection status in 372 desensitized and 528 nonsensitized patients. Factors attributed to the low viremia include posttransplant antiviral prophylaxis, PCR monitoring the presence of memory T cells, viral specific antibodies, antiviral antibodies in IVIg, and NK cell-mediated ADCC in spite of the depletion of lymphocytes. M. Cioni et al. in Ginevri's group (*IRCCS Istituto G. Gaslini, Genova, Italy*) documented that the time interval from transplant to the occurrence of DSA may influence graft injury, using longitudinally collected sera of a cohort (*n* = 114) of pediatric renal graft recipients. De novo DSA developed within a year (*n* = 15), after a year (*n* = 24), and 24.6 months (*n* = 39) posttransplant. When comparing parameters such as C1q/C3d-binding, it was noted that only younger patients developed DSA earlier. Late antibody-mediated rejection occurred in 47% of the early group and in 58% of the late-onset group. Monitoring HLA antibodies throughout the posttransplant course was emphasized, despite its high costs and organizational challenges.

V. Jucaud (*Terasaki Foundation Laboratory, Los Angeles, CA, USA*) hypothesized that comparing the highest predicted binding affinity of nonself and self allo-HLA peptide for a transplant recipient's HLA-II antigens may distinguish immunogenic (which induces DSA formation) from nonimmunogenic mismatches. This hypothesis was tested on six renal-allograft recipients with HLA-II mismatches using different programs (HLA-matchmaker, PIRCHE-II, and an HLA-II immunogenicity algorithm). A significant association between DSA formation and the predicted HLA-DR presentation of nonself peptides was noted. It was shown that the methodology predicted DSA formation based on HLA mismatches, the recipients' HLA-DR phenotypes, and their identified permissible HLA mismatches to optimize HLA matching and to guide donor selection. K. Geneugelijk et al. in Spierings's group (*University Medical Center Utrecht, Utrecht, The Netherlands*) validated the computational methodology that they developed, which uses HLA haplotype frequency to allow epitope-based HLA matching from serological split level HLA typing. Their data documented that their computational approach is a powerful and reliable tool to estimate PIRCHE-II and epilet values when high-resolution HLA genotype data is not available.

The following two articles fall into the category of clinical studies: L. Zhu et al. (*Tongji Hospital, Huazhong University of Science and Technology, Wuhan, China*) reported the incidence and patterns of early acute rejection episodes and a year of graft and patient survival outcomes of 33 renal allograft recipients after the 2nd, 3rd, and 4th kidney retransplants performed at their hospital. They documented a low incidence of early acute antibody-mediated rejection and satisfactory survival of the organ and patients after a year. The retransplant recipients had a high risk of developing early acute T cell-mediated rejection. The need for accurate diagnosis and reliable suppressive strategy was emphasized. N. Lachmann et al. (*Charite Universitatsmedizin Berlin, Berlin, Germany*) documented that 5-year graft survival, graft function, DSA levels, and serum creatinine levels can be improved with stepwise modification of the treatment regimens in a cohort (*n* = 12) with biopsy-proven antibody mediated rejection. The allograft recipients were initially administered (for 3 to 4 years) with rituximab/low-dose IVIg and plasmapheresis, then with bortezomib/low-dose IVIg (for a year) and later with bortezomib, high-dose IVIg, and plasmapheresis (> a year). These patients exhibited a significant reduction in serum creatinine and anti-HLA DSA after high-dose IVIg.

In addition, there are several review articles submitted for this special issue. E. J. Filippone and J. L. Farber (*Thomas Jefferson University, Philadelphia, USA*) reviewed the role of epitope analysis in optimizing HLA matching and assessed the pathogenicity of HLA antibodies in renal transplantation. E. Y. Cheng (*Terasaki Foundation Lab. & University of California, Los Angeles, CA, USA*) compared the humoral responses to allografts in liver and renal transplantation. In particular, the need for defining the histopathological characteristics of antibody-mediated liver graft rejection was emphasized and the question of whether all HLA antibodies are pathogenic in transplantation was addressed. A. Bharat and T. Mohanakumar (*NW Feinberg School of Medicine, Chicago, St. Joseph's Hospital and Medical Center, Phoenix, AZ, USA*) critically examined the role of humoral responses to tissue-restricted non-HLA self-antigens (such as AT1R, collagen V, and k-a1 tubulin) in lung allograft survival in recipients' posttransplantation. C. Lefaucheur et al. (*INSERM, UMR-S970, Paris, France*) reviewed risk stratification of renal allograft rejection-based factors related to anti-HLA DSA antibodies, such as antibody strength, complement-binding capabilities, and IgG subclasses. They pointed out the identification of specific allograft injury patterns based on the nature of HLA-DSA, which may elucidate therapeutic strategies such B cell depletion or complement blockade with C5 inhibitors or C1 inhibitors. S. Sethi et al. with Jordan's group (*Cedars-Sinai Medical Center, Los Angeles, CA, USA*) reviewed the agents, IVIg, anti-CD20 antibodies (rituximab, obinutuzumab), proteasome inhibitors (bortezomib, carfilzomib), anti-IL-6R blocker (tocilizumab), IgG endopeptidase (Ides® produced by *Streptococcus pyogenes)*, blockers of B cell stimulator protein to B cell receptor (belimumab) blockers of C5 to inhibit the complement sequence (eculizumab), C1 esterase inhibitor, and belatacept (CTLA-Ig that can inhibit plasma cells and DSA generation) used in desensitization. S. Wang et al. in Zhu's group (*Fudan University, Shanghai, China*) summarized both antiendothelial alloantibodies (preformed and de novo antibodies against ABO blood groups, HLA, MICA) and autoantibodies (preformed and de novo antibodies against AT1R, ETAR, Perlecan, de novo against vimentin and preformed against endoglin, FLT3 ligand, and EDIL3). The alloantibodies are implicated in hyperacute and acute rejections and long-term graft injury, whereas the autoantibodies are implicated in acute and chronic graft injury. It is emphasized that endothelial cells are indispensable participants in the pathophysiology of antibody-mediated rejection, and therapies targeted at them show promise as an improvement over the prevailing immunosuppressive modalities. C. L. Butler et al. in Reed's group (*David Geffen School of Medicine, University of California, Los Angeles, CA*) identified microvasculature as the principal target of antibody-mediated injury and illustrates that both DSA and non-DSA are pathogenic through multiple mechanisms that have extensive crosstalk, such as T cell activation by antiendothelial antibodies. Since allograft rejection can occur throughout the lifetime of a transplanted organ, this study emphasized the need to delineate the crosstalk between HLA and non-HLA antibodies and their synergistic effects on graft injury and to assess their incidence among organ types. N. El-Awar et al. (*Terasaki Foundation Laboratory, Los Angeles, CA 90064, USA*) attributed the phenomenon of cross-reactivity in HLA antibodies to shared epitopes among HLA antigens and summarized the well-defined HLA-I unique epitopes, including cryptic epitopes, on *β*2-microglobulin-free HLA and HLA-II epitopes. It is suggested that the epitope-based matching for donor organs would minimize de novo DSA, improve allograft survival, and protect the allograft against chronic rejection. C. Süsal et al. (*University of Heidelberg, Heidelberg, D-69120, Germany*) reviewed the “Heidelberg algorithm,” different measures of which may include monitoring donor-independent and donor-dependent antibodies to HLA class I and II, CDC T cell cross-matches in first transplants and B cell cross-matches in retransplant patients, having MFIs > 1000 using Luminex Labscreen Beadset in living donor organ recipients, having soluble CD30 ≥ 80 ng/ml, having good HLA match positivity in diseased donor organ recipients, acceptable mismatches from the Eurotransplant program, triple immunosuppressive regimens, protocol biopsies on days 7 and 90, DSA monitoring on days 0, 7, 30, 180 and every six months thereafter and running C1Q assays if DSA ≥ 3000 MFI. It is proposed that the algorithm has the potential to increase the number of transplantations in high-risk presensitized patients and diminishing (not totally eliminating) the impact of pre-existing antibodies on graft survival. It is hypothesized that T cell help from a preactivated immune system supports the deleterious impact of pretransplant DSA that would otherwise disappear in many graft recipients. M. H. Ravindranath (*Terasaki Foundation Laboratory, Los Angeles, CA 90064, USA*) reviewed the findings on the anti-HLA-E IgG2a mAbs (TFL-006 and TFl-007) and how they reacted with several HLA-Ia and HLA-Ib antigens, similar to that of therapeutic IVIg. In vitro, the mAbs mimicked IVIg in suppressing both antigen-specific activated T cells and anti-HLA Ab production by activated B cells, and they also expanded CD4+, CD25+, and Foxp^3^+ Tregs, which are known to suppress T and B cells involved in antibody production. Therefore, it is proposed that the humanized version of mAbs would be useful in lowering Abs in allograft recipients in sensitized patients, promoting graft survival and preventing and controlling autoimmune diseases. M. Hamdorf et al. (*Terasaki Foundation Laboratory, Los Angeles, CA, USA*) discussed whether circulating miRNA, found in serum, plasma, and urine, can serve as an alternate to invasive biopsies and can function as an early noninvasive and robust diagnostic biomarker. This can help to develop further insight into pathways leading to the rejection process and to predict allograft rejection and failure.

In summary, this special issue encompasses advances in basic, clinical, and therapeutic aspects of the IgG antibodies existing in patients waiting for donor organs and for those who experience graft injury and loss after transplantation. The contributing authors highlighted the importance of identifying anti-HLA DSA that are pathogenic to allografts and the challenges encountered in monitoring the antibodies. Some investigators indicate that the role of antibodies may encompass cell mediated immune responses. Many authors consistently highlight the challenges encountered and the need for systematic, randomized controlled clinical trials for developing appropriate therapies to downregulate antibodies or antibody subclasses truly pathogenic to the allograft. As the late Professor Terasaki stated, “The purpose of a theory is to stimulate research proving its validity” (p.669, *AJT*, 2003, 3). We hope that this special issue stimulates further research to prove or disprove the validity of the humoral theory of rejection.

*Mepur H. Ravindranath* *Junchao Cai* *Soldano Ferrone* *Frans H. J. Claas* *Senthamil R. Selvan*

